# Chemokine and Cytokine Profiles in Patients with Hand Osteoarthritis

**DOI:** 10.3390/biom11010004

**Published:** 2020-12-22

**Authors:** Jiří Baloun, Tereza Kropáčková, Hana Hulejová, Michal Tomčík, Olga Růžičková, Olga Šléglová, Jindřiška Gatterová, Jiří Vencovský, Karel Pavelka, Ladislav Šenolt

**Affiliations:** 1 Institute of Rheumatology, 128 00 Prague, Czech Republic; baloun@revma.cz (J.B.); kropackova@revma.cz (T.K.); hulejova@revma.cz (H.H.); tomcik@revma.cz (M.T.); ruzickova@revma.cz (O.R.); sleglova@revma.cz (O.Š.); gatterova@revma.cz (J.G.); vencovsky@revma.cz (J.V.); pavelka@revma.cz (K.P.); 2 Department of Rheumatology, 1st Faculty of Medicine, Charles University, 128 00 Prague, Czech Republic

**Keywords:** hand osteoarthritis, erosive disease, cytokines, chemokines

## Abstract

Background: The development of hand osteoarthritis (HOA) and its progression into the erosive subset are unclear, but inflammation is suspected to be the main source. To verify the involvement of inflammation in HOA pathogenesis, we evaluate serum inflammatory mediators and their association with HOA-related clinical features in patients. Methods: 153 participants (50 non-erosive HOA patients, 54 erosive HOA patients, and 49 healthy control subjects) were included in this study. All patients underwent clinical examination, which included assessment of tender and swollen small hand joints, ultrasound (US) examination, and self-reported measures (e.g., AUSCAN or algofunctional indexes). Serum inflammatory mediators were quantified using human cytokine 27-plex immunoassay. We employed linear modelling, correlation analysis, and resampling statistics to evaluate the association of these mediators to HOA. Results: We identified increased levels of nine inflammatory mediators (e.g., eotaxin, monocyte chemoattractant protein 1, interleukin-8, and tumour necrosis factor) in HOA patients compared to healthy controls. Increased mediators correlated with ultrasound findings as well as with clinically tender and swollen joint counts in patients with erosive HOA. However, none of the mediators distinguished between erosive and non-erosive HOA subtypes. Conclusion: Our findings support the hypothesis on the involvement of inflammation in HOA.

## 1. Introduction

Hand osteoarthritis (HOA) is the most common articular disease that causes pain and functional impairment [1,2]. The symptoms of HOA occur in approximately 6–8% of adults, with increasing prevalence during ageing and in females, and radiographic signs are found in up to two-thirds of females over 55 years of age [3]. The progression of HOA is slow but consistently leads to the loss of hyaline cartilage, changes in the subchondral bone, and osteophyte formation. Although HOA is considered a mild condition, a transient flare of the disease is often associated with inflammatory irritation of synovial tissue and pain [4].

Erosive HOA is a subset of HOA associated with more severe clinical symptoms than the non-erosive subtype [5]. The process leading to erosive changes in HOA is still unknown; however, increasing evidence indicates that inflammation is one of the substantial driving forces [6]. In fact, ultrasound (US) and magnetic resonance imaging (MRI) frequently detect synovial inflammation with structural progression and erosion development in patients with erosive HOA [7,8]. Moreover, synovial inflammation is more frequent in patients with erosive than in non-erosive HOA, which has been observed not only in erosive but also in non-erosive joints [9]. All these findings have led to the assumption that OA development and erosive evolution are the results of low-grade inflammation [10].

In general, chemoattraction and regulation of recruited immune cells, mediated by chemokines, cytokines, and growth factors, play a crucial role in OA pathophysiology and disease progression [11]. However, only a few studies have addressed the correlation between serum cytokines and clinical measures of OA. For example, interleukin (IL)-6, macrophage colony-stimulating factor (MCSF)-1, fibroblast growth factor (FGF)-21, and tumour necrosis factor-related weak inducer of apoptosis (TWEAK) have been independently associated with pain intensity in patients with knee OA [12]. In addition, plasma levels of CCL-3 and CCL-4 chemokines were correlated with the severity of radiographic damage in patients with knee OA [13]. In patients with erosive HOA, the levels of C-reactive protein (CRP) and myeloperoxidase were higher compared to those with non-erosive HOA, but the levels of CRP could not distinguish HOA patients from healthy subjects, nor did they correlate with erosive disease, structural damage, or inflammation [14,15].

However, as far as we know, no published study has shown the cytokine profile and its correlation with clinical assessments of disease severity in patients with HOA. Therefore, the present study aims to explore the serum levels of systemic mediators in patients with HOA and their association with OA-related clinical features and to elucidate the potential difference between erosive and non-erosive disease compared to healthy controls using a serum 27-plex assay of human cytokines, chemokines, and growth factors.

## 2. Materials and Methods

### 2.1. Patients

Altogether, this study included 153 subjects who met selection criteria; their sera were stored in the biobank of the Institute of Rheumatology (Prague, CZ). One hundred and four patients met the American College of Rheumatology (ACR) classification criteria for HOA [16], and they consecutively attended the outpatient department at the Institute of Rheumatology in Prague. Out of them, 50 patients were diagnosed with the non-erosive form and 54 with the erosive form. We did not apply any sampling method to balanced the sample size between subgroups. As healthy controls, 49 subjects from the biobank were selected. Control subjects were age- and sex-matched to both HOA groups, and they were without joint pain or clinical signs of HOA. CRP levels and inflammatory mediators were also evaluated in healthy control subjects. The exclusion criteria for both HOA patients and controls were the presence of systemic inflammatory disease or cancer.

Clinical examinations were performed by qualified rheumatologists. The number of clinically tender and swollen joints was recorded. Pain, stiffness, and function were assessed by the Australian/Canadian (AUSCAN) hand osteoarthritis index [17]. Hand disability was evaluated based on the algofunctional index [18]. The visual analogue scale (VAS) and the health assessment questionnaire (HAQ) were used for the assessment of pain and function/disability. Written informed consent from each subject was obtained prior to enrolment, and the study was approved by the local ethics committee at the Institute of Rheumatology in Prague, Czechia (approval number 5675/2015).

### 2.2. Radiographs

Radiographs of both hands were examined by an experienced radiologist who was blinded to the patients’ clinical examination and laboratory results. Erosive HOA was defined by at least one interphalangeal joint with radiographic signs of erosion. Radiographs of knees and hips were performed to screen the presence of OA in other locations using the Kellgren–Lawrence and Kallman scoring systems for all patients [19,20].

### 2.3. Ultrasound

Ultrasound of all joints of both hands for the assessment of power Doppler (PD) and greyscale (GS) synovitis was performed by two ultrasonographers using Esaote Mylab 60 equipment (Esaote S.p.A., Genova, Italy) and a linear transducer with an 18 MHz frequency, as described elsewhere [21]. Synovitis in GS and PD were scored semiquantitatively (0–3). The ultrasonographers were blinded to the patients’ clinical examination and laboratory results. Inter- and intraobserver reliabilities have recently been published, with moderate to very good results; Cohen’s kappas for inter- and intraobserver reliabilities ranged from 0.527 to 0.963 [22]. 

### 2.4. Laboratory Measurements 

Peripheral blood samples were collected from all subjects, processed to isolate blood serum, and stored at −80 °C for Luminex-based analysis. CRP levels were quantified turbidimetrically using the Beckman Coulter AU system (Beckman Coulter, Brea, CA, USA). 

### 2.5. Luminex Technology

For all measurements, fluorescent-bead-based instrument Bio-Plex 200 (Bio-Rad, Hercules, CA) was used according to the manufacturer’s protocol. Data were acquired on a validated and calibrated Bio-Plex 200 system (Bio-Rad) and analysed with Bio-Plex Manager 6.1 software (Bio-Rad), with a detection target of 50 beads per region, low RP1 target for CAL2 calibration, and recommended doublet discriminator (DD) gates of 5000 (Low) and 25,000 (High).

The serum levels of cytokines, chemokines, and growth factors were determined using a Bio-Plex Pro™ Human Cytokine 27-plex Assay, Group I (Bio-Rad). The kit measures the concentration of FGF basic, eotaxin, granulocyte colony-stimulating factor (G-CSF), granulocyte-macrophage colony-stimulating factor (GM-CSF), interferon-γ (IFN-γ), IL-1β, IL-1RA, IL-2, IL-4, IL-5, IL-6, IL-7, IL-8, IL-9, IL-10, IL-12 (p70), IL-13, IL-15, IL-17A, interferon gamma-induced protein (IP)-10, monocyte chemoattractant protein (MCP)-1 (MCAF), macrophage inflammatory proteins (MIP)-1α, MIP-1β, RANTES, tumour necrosis factor (TNF), and vascular endothelial growth factor (VEGF). All reagents were applied and prepared according to the manufacturer’s protocol (#10014905). Measurements were done with the selected region of specific beads. Samples were measured in duplicate and the mean value of the duplicates was taken. Cytokine and chemokine concentrations were calculated by reference to the standard curve. All samples were measured in one run (without batch effect).

### 2.6. Statistical Analyses

Statistical analyses were performed using the R programme [23] and the extended R packages [24,25,26,27]. Non-normally distributed data of mediators were log10-transformed (log-transformed) for multiple linear modelling (LM) analyses. Group comparisons with regard to healthy controls, both erosive and non-erosive HOA, additional joint OA, and disease-related clinical scales were analysed using multiple LM analysis or logistic regression, including age, gender, CRP, and BMI as confounders. Multiple comparisons were adjusted by Bonferroni’s correction. The effect size was calculated as partial omega. ANOVA was used to assess the difference and *p*-value >0.05 suggested no difference between/among groups. Permutation and bootstrapped tests utilised nontransformed data to calculate *p*-values and medians of mediator concentrations with 95% confidence intervals (CIs), respectively, in 50,000 simulations (resampling). Mediators without overlapping 95% CIs and *p*-values < 0.05 were considered different between the two diagnostic groups. 

Correlation analyses of inflammatory mediators and clinical manifestations were performed on non-erosive and erosive HOA patients separately. Since the majority of mediator concentrations and clinical manifestations revealed non-normal distribution and no transformation improved the normality, we employed Kendall’s tau coefficients. *p*-value <0.05 and r_τ_ <-0.3 or >0.3 suggest an association between the mediator and the clinical manifestation.

## 3. Results

### 3.1. Characteristics of the Study Population

The demographic and clinical characteristics of all the individuals are summarised in Table 1. Altogether, our study included 153 subjects, out of whom 54 were patients with erosive HOA, 50 were patients with the non-erosive disease, and 49 were healthy controls. In agreement with the gender proportion of the disease in the population, our cohort included significantly more female subjects (χ2 = 80.52, *p* < 0.001; 87%), but the test of proportion did not suggest any difference among groups (χ2 = 0.04, *p* = 0.979). Patients with HOA were older than controls (χ2 = 4.0, *p* = 0.045), but age was likely insignificant (*p* > 0.05) among non-erosive, erosive and control groups after pairwise comparison with Bonferroni’s correction. The difference in BMI was negligible among groups. Most of the patients (89%) were taking symptomatic slow-acting drugs (SYSADOA) twice a year, nonsteroidal anti-inflammatory drugs (NSAIDs), or analgesics on demand.

Clinical assessment parameters of hand stiffness defined by AUSCAN stiffness (χ2 = 0.45, *p* = 0. 505), functional limitation defined by AUSCAN function (χ2 = 2.54, *p* = 0.111), and the sum of AUSCAN indexes defined by the AUSCAN total (χ2 = 3.61, *p* = 0.058) were without difference between patients with erosive and non-erosive HOA. In addition, the number of clinically tender (χ2 =2.91, *p* = 0.088) and swollen (χ2 =1.77, *p* = 0.183) joints, VAS score (χ2 = 0.62, *p* = 0.433), and HAQ index (χ2 = 0.091, *p* = 0.763) were comparable between both subgroups of HOA in our cohort. On the contrary, hand pain defined by AUSCAN pain (χ2 = 3.89, *p* = 0.049) and the algofunctional index (χ2 = 5.33, *p* = 0.028) were probably different between non-erosive and erosive disease. US-detected pathologies such as grey scale (GS) synovitis (χ2 = 28.67, *p* < 0.001) and the intensity of the power Doppler (PD) signal (χ2 = 18.93, *p* < 0.001) were significantly more pronounced in patients with erosive compared with non-erosive disease. 

### 3.2. Inflammatory Mediators are Elevated in Patients with Hand Osteoarthritis 

The applied human cytokine assay for Luminex is predesigned to detect and quantify 27 cytokines, chemokines, and growth factors. We chose this method for the simultaneous detection of multiple cytokines, simple interpretation of results, and minimal sample volume for analysis. However, 12 molecules were undetectable due to their low serum concentrations in both patients and healthy controls. 

**Figure 1:**
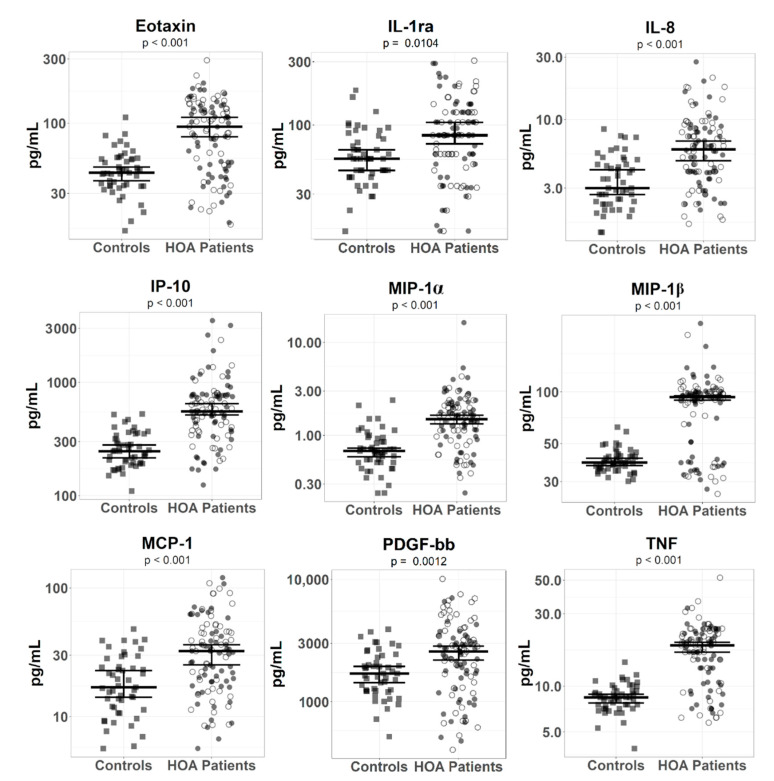
Serum levels of inflammatory mediators in patients with hand osteoarthritis and in healthy controls. The error bars represent 95% confidence intervals with the median calculated by a bootstrapped method with 50,000 simulations for healthy controls and HOA patients. *p*-values (depicted below mediators’ names) show statistical significance between healthy controls and HOA patients and were computed by the permutation test with 50,000 simulations. Healthy controls and erosive and non-erosive HOA patients are depicted as filled squares and filled and unfilled circles, respectively.

Since the raw data of mediator levels in sera had non-normal distribution, we log-transformed data before multiple LM analyses with confounders, which identified 11 mediators with significantly higher serum concentrations between patients with HOA and healthy controls (Table 2A). Out of these significantly different mediators, eotaxin (F = 33.48, *p* < 0.001), IL-8 (F = 28.63, *p* < 0.001), IP-10 (F = 51.65, *p* < 0.001), MIP-1α (F = 41.74, *p* < 0.001), MIP-1β (F = 67.71, *p* < 0.001), and TNF (F = 67.93, *p* < 0.001) had an effect size over 0.14, indicating substantial differences between HOA patients and healthy controls (Figure 1). Other significantly elevated mediators in patients included MCP-1 (F = 18.17, *p* < 0.001), PDGF-bb (F = 7.33, *p* < 0.001), IL-1RA (F = 4.65, *p* < 0.001), and IL-1β (F = 4.57, *p* = 0.026), but they had an effect size below 0.14, suggesting a small association between the groups. IL-17 was significantly downregulated in HOA patients (F = 7.48, *p* = 0.007), with an effect size of 0.049, indicating negligible difference.

In order to validate the output of the statistical analysis with log-transformed data, we employed a different statistical approach—resampling statistics (permutation and bootstrapped tests; Table S1A)—that belongs to nonparametric tests and does not require the assumption of normal distributions. This additional analysis revealed 11 mediators with *p* < 0.05, but IL-17 had overlapping CIs between HOA patients and healthy controls, indicating no statistical difference between groups, and was discarded in the subsequent selection. To sum up, this analysis confirmed the significant difference of the nine mediators (eotaxin, IL-1RA, IL-8, IP-10, MCP-1, MIP-1α, MIP-1β, PDGF-bb, and TNF) detected in the previous statistical analysis.

### 3.3. Inflammatory Mediators in Non-Erosive and Erosive Subgroups of Hand Osteoarthritis

In order to identify mediators that are different between erosive and non-erosive HOA, we subclassified patients into erosive and non-erosive HOA subgroups. This analysis revealed no significant difference between mediators in both HOA subtypes with *p* < 0.05 (Table 2B). 

The permutation and bootstrapped tests revealed that medians of MCP-1 and RANTES were different between erosive and non-erosive HOA with *p*-value < 0.05, but their overlapping 95% CIs indicated no difference (Table S1B). No other mediator demonstrated differences between erosive and non-erosive HOA.

### 3.4. The Correlation between Inflammatory Mediators and Clinical Manifestations 

Kendall’s correlation analysis detects the strength and direction of the association between mediators and clinical symptoms of HOA, and we considered correlations with *p* < 0.05 and r_τ_ <−0.3 or >0.3 significant. This analysis revealed that US pathologies and clinically swollen and tender joint counts had the highest association to studied mediators (Table 3). All analysed correlations between mediators and clinical manifestations are shown in Table S2, including correlation coefficients and *p*-values.

In patients with erosive HOA, we found close correlations between mediators and clinical manifestations (Table 3B). In brief, eotaxin, IL-1RA, IL-8, IP-10, MCP-1, MIP-1α, MIP-1β, PDGF bb, RANTES, and TNF were positively correlated with GS synovitis and the number of GS-positive joints, respectively. Out of these mediators, eotaxin, MCP-1, MIP-1β, and TNF were also correlated with clinically tender and swollen joint counts. On the contrary, IL-17 was negatively correlated with GS synovitis and the number of PD-positive joints, with *p* < 0.05, but correlation coefficients were −0.22 and −0.24, respectively (Table 3B).

The correlation analysis between mediators and clinical features in non-erosive HOA patients suggested, however, a negative correlation of MCP-1 with GS synovitis (r_τ_ = −0.36, *p* < 0.001) and the number of GS-positive joints (r_τ_ = −0.34, *p* = 0.001). Moreover, this analysis indicated a negative correlation of eotaxin, MIP-1α, RANTES, and PDGF-bb with GS synovitis and the number of GS-positive joints, but their correlation coefficients were between −0.3 and −0.25 (Table S2A). CRP weakly correlated only with IP-10 (r_τ_ = 0.26 *p* = 0.007). Our analysis of non-erosive HOA disease did not suggest any correlation of mediators with clinically tender or swollen joint counts, which are considered predominant symptoms of HOA. 

### 3.5. Osteoarthritis at Additional Joint Sites and Its Influence on the Levels of Inflammatory Mediators

Since the presence of OA at other sites might influence systemic cytokine levels in HOA patients, we additionally divided HOA patients based on the presence of hip and knee OA into (a) patients with hip OA, (b) patients with knee OA, (c) patients with both hip and knee OA, and (d) patients without hip or knee OA. This comparison analysis did not reveal any statistical differences between patients with and without OA at other sites (Table S3).

## 4. Discussion

In this study, we provide an extensive profile analysis of serum mediators and their association with OA-related clinical features in patients with HOA. We found a significant increase of nine mediators in patients with HOA compared to healthy controls. Additionally, we observed considerable correlations between these mediators, clinically tender and swollen joints, as well as US-detected pathologies in patients with erosive HOA. 

Erosive HOA is considered to be more severe than the non-erosive subset; it mostly manifests worse clinical symptoms such as pain, functional impairment, and synovial inflammation [28]. In agreement, we found a significantly worse algofunctional index and worse US-detected pathologies, reflecting the role of inflammation in erosive HOA; however, probably due to the low number of patients, the number of tender and swollen joints did not significantly differ between the erosive and non-erosive subsets. Although increased serum CRP levels were previously found in erosive HOA [29], recent studies [14,30] and our results did not confirm CRP elevation in erosive compared to non-erosive HOA patients. In addition, CRP levels were not different between HOA patients and healthy controls. 

However, to our knowledge, this is the first study showing the elevation of systemic chemokines and cytokines in HOA patients. Using the human cytokine assay, we profiled 27 mediators in sera of HOA patients and employed two statistical approaches to determine their potential clinical relevance. Based on the criteria of (a) *p*-value <0.05 and (b) nonoverlapping CI, we found nine serum mediators (eotaxin, IL-1RA, IL-8, IP-10, MIP-1α, MIP-1β, MCP-1, PDGF-bb and TNF) that were significantly elevated in HOA patients compared to healthy controls. Our findings are consistent with other studies showing that increased production of proinflammatory and chemoattractive mediators is associated with the process of OA pathology [31,32,33,34]. The majority of the elevated chemokines in patients’ sera (e.g., IL-8, MCP-1, MIP-1α, or eotaxin) play a pivotal role in the chondrocyte activation and amplification of the inflammatory process in OA [11,35,36]. These chemokines and cytokines recruit immune cells [37,38,39,40] into the affected joints and can diffuse into the bloodstream; they might also assist in better understanding of the biological background of the disease process.

Oliviero et al. [41] recently found higher levels of cytokines IL-1, IL-6, and IL-8, as well as matrix-degrading enzymes matrix metalloproteinase (MMP)-1 and MMP-3, in the synovial fluid of patients with knee OA and concomitant erosive HOA compared to those with the non-erosive disease. Although synovial fluid is an ultrafiltrate of plasma and systemic elevation of inflammatory markers could be expected, the levels of mediators were similar between both HOA subsets in our study. This inconsistency between synovial fluid and plasma could be explained by the site-specific function of mediators, or, in addition to inflammatory cytokines, other mechanisms could be responsible for the development of erosive changes. On the other hand, we were able to demonstrate a positive correlation of the nine aforementioned elevated mediators and RANTES with GS synovitis and the number of GS-positive joints, particularly in patients with erosive HOA. Altogether, the elevated levels of mediators and their association with clinical features of HOA support the hypothesis on the involvement of inflammation in the process of HOA and the potential progression into the erosive disease [11,35,36,42].

All nine increased mediators of inflammation have been previously validated as key players in modulating the immune response of various rheumatic diseases, such as rheumatoid arthritis (RA), spondyloarthritis, and systemic lupus erythematosus [43,44,45]. Therefore, based on the role of inflammation in the pathogenesis of OA [10], our results support the contribution of inflammation to the development and progression of HOA.

Two of the nine dysregulated mediators found in this study have been previously targeted by monoclonal antibodies in patients with OA to assess their efficacy. Anti-TNF targeting agents are successfully used in the treatment of inflammatory rheumatic diseases, such as RA and spondyloarthritides (review in Tylor et al.) [46]. However, the disease modification effect of adalimumab (anti-TNF monoclonal antibody) has not been proven in patients with hand OA [47,48]. Similarly, the inhibition of the important proinflammatory mediator IL-1 by lutikizumab did not improve pain or imaging outcomes in patients with erosive HOA compared with placebo [49]. In addition, diacerhein, which also reduces IL-1, was not effective in controlling the symptoms of hand OA [50]. Altogether, since targeting TNF and IL-1β have not been successful in hand OA, other dysregulated mediators in this study may represent potential therapeutic targets for OA treatment.

Interestingly, we found weak negative correlations of eotaxin, IP-10, MCP-1, MIP-1α, PDGF-bb, RANTES, and TNF with GS synovitis in patients with non-erosive HOA. We cannot explain these opposite correlations and only speculate that the immune system in these patients dampens the mediators’ activities with increasing HOA manifestation. In contrast, the immune system of patients with erosive HOA mistakenly amplifies the mediators’ activities, which probably leads to further joint damage or additional proinflammatory mechanisms to costimulate the mediators’ secretions. However, this hypothesis has to be validated in further studies.

However, there are some limitations to this study. First, the number of analysed samples was relatively small, and it was difficult to balance all confounding factors or apply advanced biostatistical approaches. Second, the applied human cytokine 27-plex assay did not reach the ELISA`s sensitivity and undetected inflammatory mediators might still play a role, although they were below the detection threshold. Third, the presence of erosive HOA was examined using the Kellgren–Lawrence and Kallman scoring systems; other scoring systems might result in different patient categorisation. Finally, this study is descriptive, and our results have to be validated by additional research.

## 5. Conclusions

We found systemic elevation of nine mediators in both erosive and non-erosive HOA patients compared to healthy subjects. Additionally, these mediators positively correlated with clinical and imaging findings of patients with the erosive subset of HOA. These findings support the active role of inflammation in the process of HOA. However, further studies validating our findings and longitudinal data are needed.

## Figures and Tables

**Table 1:** Baseline characteristics of patients with hand osteoarthritis and control subjects. The data are presented as mean (the standard deviation; SD) for normal distribution or median (interquartile range; IQR) for deviated distribution. Logistic regression was applied to assess the difference in clinical manifestations between non-erosive and erosive disease. Gender, age, CRP, and BMI served as confounders in the model. *p*-values were computed using ANOVA. *** *p* < 0.001; * *p* < 0.05.

	HOA Patients (n = 104)	Patients with Non-Erosive HOA (n = 50)	Patients with Erosive HOA (n = 54)	Healthy Controls (n = 49)
Female, n (%)	90 (87)	43 (86)	47 (87)	42 (86)
Age [years], Mean ± SD	66.3 ± 8.2	64.3 ± 7.3	67.6 ± 8.6	62.6 ± 8.2
CRP [mg/L], Median (IQR)	3.2 (1–3.5)	2.9 (0.9–3)	3.4 (1.1–3.5)	2.3 (0.9–3)
BMI, Mean ± SD	27.0 ± 3.5	26.6 ± 3.9	27.4 ± 4.3	26.4 ± 3.7
Clinically tender joints [count], Median (IQR)	6 (2–9)	5 (2–8)	6 (2–9)	-
Clinically swollen joints [count], Median (IQR)	5 (1–7)	5 (17)	5 (1–7)	-
Algofunctional index, Median (IQR)	19 (14–22)	17 (13–21)	19 (14–24) *	-
AUSCAN—total, Mean ± SD	22.5 ± 10.4	20.3 ± 9.1	23.9 ± 11	-
AUSCAN—pain, Mean ± SD	8.4 ± 4.2	7.5 ± 3.8	9 ± 4.4 *	-
AUSCAN—stiffness, Mean ± SD	1.9 ± 0.9	1.9 ± 0.9	2 ± 0.9	-
AUSCAN—function, Median (IQR)	12 (6–17)	10.9 (6–15.8)	12.8 (7–18)	-
VAS, Median (IQR)	44.6 (28–59)	42.2 (30–54.5)	46.7 (26–67)	-
HAQ, Median (IQR)	0.8 (0.4–1.3)	0.8 (0.4–1.1)	0.9 (0.4–1.3)	-
GS synovitis—total, Median (IQR)	7.5 (1–12.5)	5 (0–5.5)	9.3 (2–15) ***	-
GS synovitis—joint count, Median (IQR)	2 (0–3)	1.4 (0-2)	2.5 (0–4) ***	-
PD synovitis—total, Median (IQR)	5 (1–8)	4 (0–6)	7 (2–11) ***	-
PD synovitis—joint count, Median (IQR)	2 (0–3)	1 (0–2)	2 (0–3) ***	-
Knee OA [count] n (%)	30 (29)	12 (24)	18 (33)	-

Abbreviations: AUSCAN, Australian/Canadian osteoarthritis hand index; VAS, visual analogue scale; HAQ, health assessment questionnaire; CRP, C-reactive protein; OA, osteoarthritis; HOA, hand OA; GS, greyscale; PD, power Doppler.

**Table 2:** Serum levels of mediators in patients with hand osteoarthritis and healthy controls. The difference was studied between (A) HOA patients and healthy controls and (B) patients with erosive and non-erosive HOA. ANOVA was performed for both subgroups. Data were described as *p*-value, effect size (computed as partial omega) with 95% CI, and fold-change (calculated as the proportion of medians followed by log2-transformation; positive values indicate upregulation in HOA patients and patients with the erosive disease, respectively). Mediators are sorted in ascending order based on *p*-values.

A				B			
Mediators	*p*-Value	Effect Size (95% CI)	Log2fc	Mediators	*p*-Value	Effect Size (95% CI)	Log2fc
TNF	<0.001	0.314 (0.200–0.431)	1.14	RANTES	0.074	0.019 (−0.01–0.113)	0.20
MIP-1β	<0.001	0.314 (0.200–0.430)	1.27	PDGF-bb	0.123	0.012 (−0.01–0.1)	0.25
IP-10	<0.001	0.258 (0.147–0.376)	1.23	MCP-1	0.154	0.008 (−0.01–0.09)	0.49
MIP-1α	<0.001	0.218 (0.112–0.337)	1.18	Eotaxin	0.217	0.003 (−0.01–0.079)	0.42
Eotaxin	<0.001	0.182 (0.082–0.299)	1.12	TNF	0.280	0.000 (−0.01–0.072)	0.15
IL-8	<0.001	0.159 (0.064–0.275)	0.93	MIP-1α	0.382	−0.001 (−0.01–0.069)	−0.09
MCP-1	<0.001	0.105 (0.027–0.213)	0.92	IL-4	0.455	−0.005 (−0.01–0.056)	0.12
IL-17	0.008	0.049 (−0.004–0.148)	0.56	IL-9	0.555	−0.007 (−0.01–0.051)	0.11
PDGF-bb	0.009	0.042 (−0.003–0.129)	−0.49	IL-8	0.566	−0.007 (−0.01–0.047)	0.18
IL-1RA	0.029	0.025 (−0.007–0.102)	0.53	IL-1ra	0.594	−0.008 (−0.01–0.045)	0.00
IL-1β	0.03	0.032 (−0.01–0.134)	0.42	MIP-1β	0.628	−0.008 (−0.01–0.044)	−0.01
IL-9	0.244	0.003 (−0.007–0.06)	−0.27	IL-1β	0.697	−0.014 (−0.016–0.055)	−0.58
IL-4	0.398	−0.003 (−0.007–0.042)	−0.39	IL-17	0.763	−0.011 (−0.013–0.043)	−0.18
IFN-γ	0.533	−0.004 (−0.008–0.046)	0.13	IP-10	0.846	−0.009 (−0.01–0.026)	0.00
RANTES	0.671	−0.005 (−0.007–0.033)	0.63	IFN-γ	0.922	−0.011 (−0.012–0.027)	−0.29

Abbreviations: log2FC, log2-transformed ratio; HOA, hand osteoarthritis; IL, interleukin; INF, interferon; IP, interferon gamma-induced protein; MCP, monocyte chemoattractant protein; MIP, macrophage inflammatory protein; PDGF, platelet-derived growth factor; TNF, tumour necrosis factor.

**Table 3:** The correlation analyses between the levels of inflammatory mediators and clinical features of hand osteoarthritis. The correlation analysis was performed using Kendall’s correlation on (A) patients with non-erosive HOA and (B) patients with erosive HOA. Both tables contain only clinically tender and swollen joints and US pathologies and depict correlation coefficients. Significant correlation coefficients ≤ −0.3 or ≥ 0.3 and *p*-values <0.05 are in bold with asterisk (* *p* < 0.05).

A						
Mediators	Clinically Tender Joints	Clinically Swollen Joints	GS Synovitis	PD Synovitis	GS Positive Joints	PD Positive Joints
Eotaxin	−0.02	−0.03	−0.26	−0.13	−0.27	−0.14
IFN-γ	0.08	0.15	−0.06	−0.12	−0.06	−0.12
IL-17	0.10	0.14	−0.04	0.01	−0.02	0.03
IL-1β	−0.16	0.02	−0.12	−0.15	−0.13	−0.16
IL-1RA	0.04	0.03	−0.19	0.06	−0.18	0.05
IL-4	0.03	0.02	−0.11	0.02	−0.10	0.00
IL-8	0.00	−0.10	−0.07	−0.09	−0.06	−0.10
IL-9	−0.06	−0.01	−0.16	0.13	−0.16	0.14
IP-10	0.04	0.02	−0.22	−0.14	−0.20	−0.14
MCP-1	−0.10	−0.12	−0.36 *	−0.17	−0.34 *	−0.18
MIP-1α	−0.07	−0.09	−0.28	−0.16	−0.26	−0.15
MIP-β	−0.16	−0.18	−0.19	−0.06	−0.17	−0.06
PDGF-bb	−0.09	−0.07	−0.27	−0.06	−0.26	−0.06
RANTES	−0.16	−0.06	−0.29	−0.05	−0.26	−0.05
TNF	−0.16	−0.11	−0.22	−0.16	−0.19	−0.16
**B**						
**Mediators**	**Clinically Tender Joints**	**Clinically Swollen Joints**	**GS Synovitis**	**PD Synovitis**	**GS Positive Joints**	**PD Positive Joints**
Eotaxin	0.35 *	0.35 *	0.38 *	−0.03	0.43 *	0.00
IFN-γ	0.24	0.20	0.21	−0.01	0.21	0.02
IL-17	−0.10	−0.02	−0.22	−0.21	−0.19	−0.24
IL-1β	0.17	0.22	0.19	−0.09	0.23	−0.07
IL-1RA	0.23	0.26	0.35 *	0.07	0.38 *	0.12
IL-4	0.15	0.23	0.12	−0.13	0.19	−0.10
IL-8	0.23	0.20	0.30 *	−0.01	0.32 *	0.00
IL-9	0.05	0.01	0.14	−0.11	0.18	−0.09
IP-10	0.29	0.25	0.43 *	−0.01	0.45 *	0.04
MCP-1	0.30 *	0.31 *	0.40 *	0.04	0.43 *	0.06
MIP-1α	0.20	0.25	0.35 *	0.05	0.35 *	0.04
MIP-β	0.31 *	0.30 *	0.50 *	0.03	0.48 *	0.06
PDGF-bb	0.21	0.18	0.44 *	0.08	0.44 *	0.09
RANTES	0.28	0.25	0.43 *	0.10	0.43 *	0.14
TNF	0.40 *	0.32 *	0.57 *	0.00	0.57 *	0.04

Abbreviations: HOA, hand osteoarthritis; IL, interleukin; INF, interferon; IP, interferon gamma-induced protein; MCP, monocyte chemoattractant protein; MIP, macrophage inflammatory protein; PDGF, platelet-derived growth factor; TNF, tumour necrosis factor.

## Data Availability

Data is contained within the article or supplementary material.

## Supplementary Materials

The following are available online at https://www.mdpi.com/2218-273X/11/1/4/s1. Table S1: Serum levels of cytokines and chemokines in patients with hand osteoarthritis (HOA) and healthy controls. The bootstrapped method computed medians with 95% CIs and the permutation method computed *p*-values between (A) HOA patients and healthy controls and (B) patients with erosive and non-erosive HOA. Values represent medians and 95% CIs in brackets. Mediators are sorted in ascending order based on *p*-values. Table S2: Correlation analyses between the levels of inflammatory mediators and clinical factors. Kendall’s correlation analysis was performed on subjects divided into two subgroups: (A) non-erosive HOA and (B) erosive HOA patients. Both tables depict correlation coefficients and *p*-values. Both coefficients <−0.3 or >0.3 and *p*-values <0.05 for a correlation of a mediator and clinical measurement are in bold. Table S3: Osteoarthritis at additional joint sites and its influence on the levels of inflammatory mediators. The effect of OA at additional joint sites was measured in HOA patients, who were divided into (a) patients with hip OA, (b) patients with knee OA, (c) patients with both hip and knee OA, and (d) patient without hip or knee OA. The LM analysis included age, gender, BMI, and CRP as confounders. *p*-values were computed using ANOVA and effect size was calculated as partial omega with 95% CI.
