# Supplementary material for: Chemokine and Cytokine Profiles in Patients with Hand Osteoarthritis
Source: Biomolecules. 2020 Dec 22;11(1):4. doi: 10.3390/biom11010004 (PMC7822191; doi:10.3390/biom11010004)
Supplement: Supplementary file 1 [file biomolecules-11-00004-s001.pdf]

Supplementary materials

Table S1 Serum levels of cytokines and chemokines in patients with hand osteoarthritis (HOA) and healthy controls. The bootstrapped method computed medians with 95% CIs and the permutation method computed p-value between (A) HOA patients and healthy controls; and (B) patients with erosive and non-erosive HOA. Values represent medians and 95% CIs in brackets. Mediators are sorted in ascending order based on *p-values*.

A

Mediator	HOA patients	Healthy controls	p-value
Eotaxin	94.01<br>(79.24-110.49)	42.7<br>(37.29-47.18)	<0.001
IL-8	5.93<br>(4.87-6.87)	3<br>(2.68-4.14)	<0.001
IP-10	554.98<br>(516.65-649.56)	246.55<br>(216.33-280.58)	<0.001
MCP-1	32.29<br>(25.29-36.25)	16.9<br>(14.14-22.8)	<0.001
MIP-1α	1.5<br>(1.34-1.65)	0.68<br>(0.59-0.73)	<0.001
MIP-1β	93.25<br>(89.62-95.2)	38.78<br>(37.25-41.08)	<0.001
TNF	18.59<br>(16.8-19.49)	8.44<br>(7.77-8.87)	<0.001
PDGF-bb	2 581.53<br>(2 194.04-2 861.11)	1 696.74<br>(1 431.29-1 945.65)	0.001
RANTES	14 213.85<br>(13 048.65-14 853.7)	8 687.99<br>(6 705.17-1 1916.85)	0.005
IL-1RA	83.4<br>(72.1-104.66)	55.4<br>(45.38-64.87)	0.011
IL-17	2.65<br>(2.04-2.99)	3.41<br>(2.78-3.71)	0.020
IL-9	3.07<br>(2.55-3.07)	3.52<br>(2.94-4.09)	0.101
IL-4	1.38<br>(1.31-1.47)	1.24<br>(1.09-1.4)	0.147
IL-1β	0.16<br>(0.1-0.19)	0.12<br>(0.12-0.16)	0.184
IFN-γ	0.98<br>(0.92-1.36)	1.16<br>(1.04-1.4)	0.489

B

Mediator	HOA patients	Healthy controls	p-value
RANTES	13 048.65<br>(10 972.83 - 14 450.95)	14 997.73<br>(13 529.86 - 15 764.61)	0.019
MCP-1	26.45<br>(21.45 - 33.57)	37.13<br>(28.89 - 44.8)	0.037
Eotaxin	77.79<br>(51.4 - 110.49)	104.19<br>(83.98 - 121.72)	0.127
PDGF-bb	2 368.33<br>(1 929.17 - 2 744.86)	2 822.4<br>(2 221.78 - 3 806.43)	0.205
IL-8	5.54<br>(4.01 - 6.44)	6.29<br>(4.58 - 8.01)	0.394
TNF	16.8<br>(14.1 - 19.49)	18.59<br>(16.8 - 20.83)	0.42
IL-4	1.34<br>(1.22 - 1.52)	1.46<br>(1.23 - 1.63)	0.542
IL-9	2.85<br>(2.55 - 3.07)	3.07<br>(2.28 - 3.86)	0.62
IL-1β	0.17<br>(0.1 - 0.19)	0.11<br>(0.1 - 0.22)	0.659
IFN-γ	1.16<br>(0.95 - 1.36)	0.95<br>(0.54 - 1.36)	0.762
MIP-1β	93.75<br>(85.53 - 96.23)	93.06<br>(89.85 - 97.19)	0.802
MIP-1α	1.56<br>(1.29 - 1.77)	1.47<br>(1.2 - 1.65)	0.815
IL-17	2.78<br>(2.04 - 3.71)	2.46<br>(2.04 - 2.99)	0.836
IP-10	554.98<br>(495.43 - 753.35)	554.39<br>(488.85 - 642.08)	0.974
IL-1RA	83.4<br>(69.43 - 104.66)	83.4<br>(60.2 - 104.66)	1.000

Abbreviations: *HOA*, hand osteoarthritis; *IL*, interleukin; *INF*, interferon; *IP*, interferon gamma-induced protein; *MCP*, monocyte chemoattractant protein; *MIP*, macrophage inflammatory protein; *PDGF*, platelet-derived growth factor; *TNF*, tumour necrosis factor.

Table S2 The correlation analyses between the levels of inflammatory mediators and clinical factors. Kendall`s correlation analysis was performed on subjects divided into two subgroups: A) non-erosive; and B) erosive HOA patients. Both tables depict correlation coefficients and *p-values*. Both coefficients < -0.3 or > 0.3 and p-values < 0.05 for a correlation of a mediator and clinical measurement are in bold.

A

		**Algofunctional index**	**AUSCAN total**	**AUSCAN pain**	**AUSCAN stiffness**	**AUSCAN function**	**CRP**	**Clinically tender joints**	**Clinically swollen joints**	**GS positive joints**	**PD positive joints**	**GS synovitis**	**PD synovitis**
Eotaxin	r_τ	0.11	0.05	0.07	-0.06	0.16	0.01	-0.02	-0.03	-0.27	-0.14	-0.26	-0.13
	p-value	0.29	0.60	0.52	0.59	0.12	0.92	0.85	0.77	0.01	0.20	0.01	0.23
IFN-γ	r_τ	0.02	0.11	0.14	0.04	0.08	-0.02	0.08	0.15	-0.06	-0.12	-0.06	-0.12
	p-value	0.87	0.32	0.21	0.75	0.50	0.83	0.46	0.20	0.63	0.32	0.64	0.32
IL-17	r_τ	0.23	0.08	0.19	-0.08	0.06	0.04	0.10	0.14	-0.02	0.03	-0.04	0.01
	p-value	0.04	0.47	0.10	0.54	0.59	0.73	0.39	0.24	0.87	0.78	0.72	0.94
IL-1β	r_τ	0.02	0.11	0.12	-0.19	0.10	0.01	-0.16	0.02	-0.13	-0.16	-0.12	-0.15
	p-value	0.87	0.42	0.37	0.19	0.44	0.96	0.25	0.86	0.35	0.27	0.40	0.32
IL-1RA	r_τ	0.13	0.21	0.18	0.01	0.26	0.11	0.04	0.03	-0.18	0.05	-0.19	0.06
	p-value	0.21	0.04	0.08	0.94	0.01	0.28	0.73	0.74	0.09	0.65	0.08	0.59
IL-4	r_τ	0.19	0.07	0.09	-0.15	0.18	-0.06	0.03	0.02	-0.10	0.00	-0.11	0.02
	p-value	0.06	0.46	0.40	0.17	0.08	0.53	0.78	0.85	0.33	0.98	0.31	0.87
IL-8	r_τ	0.12	0.04	0.03	-0.01	0.02	-0.19	0.00	-0.10	-0.06	-0.10	-0.07	-0.09
	p-value	0.22	0.69	0.77	0.90	0.86	0.05	0.99	0.31	0.56	0.38	0.50	0.44
IL-9	r_τ	0.16	0.11	0.07	-0.10	0.17	0.03	-0.06	-0.01	-0.16	0.14	-0.16	0.13
	p-value	0.13	0.26	0.50	0.40	0.10	0.74	0.55	0.93	0.13	0.21	0.15	0.25
IP-10	r_τ	0.17	0.13	0.19	0.00	0.17	0.26	0.04	0.02	-0.20	-0.14	-0.22	-0.14
	p-value	0.08	0.19	0.06	0.99	0.09	0.01	0.71	0.83	0.06	0.19	0.04	0.20
MCP-1	r_τ	-0.01	0.07	0.08	-0.05	0.10	-0.01	-0.10	-0.12	**-0.34**	-0.18	**-0.36**	-0.17
	p-value	0.95	0.48	0.42	0.63	0.30	0.90	0.35	0.24	**0.00**	0.10	**0.00**	0.11
MIP-1α	r_τ	-0.02	0.03	0.06	-0.05	0.07	0.00	-0.07	-0.09	-0.26	-0.15	-0.28	-0.16
	p-value	0.85	0.74	0.55	0.68	0.47	0.97	0.49	0.38	0.01	0.19	0.01	0.16
MIP-β	r_τ	-0.09	-0.03	-0.06	-0.05	0.03	0.03	-0.16	-0.18	-0.17	-0.06	-0.19	-0.06
	p-value	0.38	0.74	0.52	0.63	0.80	0.74	0.12	0.08	0.09	0.60	0.07	0.60

PDGF-bb	r_τ	0.02	0.18	0.14	0.11	0.22	0.02	-0.09	-0.07	-0.26	-0.06	-0.27	-0.06
	p-value	0.81	0.07	0.17	0.33	0.03	0.81	0.39	0.52	0.01	0.58	0.01	0.60
RANTES	r_τ	0.01	0.15	0.14	0.02	0.20	-0.04	-0.16	-0.06	-0.26	-0.05	-0.29	-0.05
	p-value	0.89	0.13	0.16	0.88	0.04	0.65	0.12	0.54	0.01	0.68	0.01	0.64
TNF	r_τ	-0.01	0.04	0.06	-0.08	0.08	-0.04	-0.16	-0.11	-0.19	-0.16	-0.22	-0.16
	p-value	0.96	0.71	0.55	0.47	0.40	0.72	0.13	0.31	0.08	0.16	0.03	0.14

B

		**Algofunctional index**	**AUSCAN total**	**AUSCAN pain**	**AUSCAN stiffness**	**AUSCAN function**	**CRP**	**Clinically tender joints**	**Clinically swollen joints**	**GS positive joints**	**PD positive joints**	**GS synovitis**	**PD synovitis**
Eotaxin	r_τ	0.09	0.05	0.03	0.05	0.05	-0.15	**0.35**	**0.35**	**0.43**	0.00	**0.38**	-0.03
	p-value	0.34	0.59	0.76	0.67	0.57	0.11	**0.00**	**0.00**	**0.00**	0.96	**0.00**	0.80
IFN-γ	r_τ	0.13	0.07	-0.02	-0.01	0.13	-0.14	0.24	0.20	0.21	0.02	0.21	-0.01
	p-value	0.24	0.53	0.87	0.94	0.21	0.18	0.03	0.07	0.05	0.89	0.05	0.94
IL-17	r_τ	0.12	0.08	0.18	0.01	0.00	0.10	-0.10	-0.02	-0.19	-0.24	-0.22	-0.21
	p-value	0.29	0.49	0.11	0.95	0.97	0.36	0.40	0.84	0.09	0.04	0.05	0.07
IL-1β	r_τ	0.21	0.10	0.05	0.00	0.15	-0.05	0.17	0.22	0.23	-0.07	0.19	-0.09
	p-value	0.08	0.40	0.67	0.98	0.24	0.65	0.17	0.08	0.06	0.59	0.12	0.50
IL-1RA	r_τ	0.06	0.00	-0.01	0.03	-0.02	0.03	0.23	0.26	**0.38**	0.12	**0.35**	0.07
	p-value	0.52	0.98	0.92	0.78	0.88	0.79	0.02	0.01	**0.00**	0.24	**0.00**	0.49
IL-4	r_τ	0.14	0.07	0.04	0.08	0.07	-0.14	0.15	0.23	0.19	-0.10	0.12	-0.13
	p-value	0.15	0.50	0.65	0.48	0.46	0.13	0.12	0.02	0.05	0.34	0.20	0.20
IL-8	r_τ	0.16	0.14	0.15	0.18	0.11	0.00	0.23	0.20	**0.32**	0.00	**0.30**	-0.01
	p-value	0.10	0.15	0.13	0.09	0.25	0.96	0.02	0.04	**0.00**	0.98	**0.00**	0.89
IL-9	r_τ	0.10	-0.03	-0.04	0.02	-0.02	-0.12	0.05	0.01	0.18	-0.09	0.14	-0.11
	p-value	0.34	0.76	0.71	0.88	0.85	0.23	0.64	0.94	0.08	0.38	0.16	0.28
IP-10	r_τ	-0.03	-0.04	-0.08	-0.02	-0.03	0.02	0.29	0.25	**0.45**	0.04	**0.43**	-0.01
	p-value	0.78	0.66	0.39	0.87	0.76	0.86	0.00	0.01	**0.00**	0.69	**0.00**	0.88
MCP-1	r_τ	0.08	-0.01	-0.02	0.02	0.00	-0.22	**0.30**	**0.31**	**0.43**	0.06	**0.40**	0.04
	p-value	0.39	0.95	0.85	0.85	0.98	0.02	**0.00**	**0.00**	**0.00**	0.57	**0.00**	0.70
MIP-1α	r_τ	0.06	0.06	0.05	0.10	0.06	-0.03	0.20	0.25	**0.35**	0.04	**0.35**	0.05
	p-value	0.56	0.50	0.61	0.36	0.53	0.78	0.04	0.01	**0.00**	0.72	**0.00**	0.64

MIP-β	r_τ	0.01	0.01	0.00	0.00	0.01	-0.11	**0.31**	**0.30**	**0.48**	0.06	**0.50**	0.03
	p-value	0.90	0.90	0.99	0.97	0.95	0.25	**0.00**	**0.00**	**0.00**	0.56	**0.00**	0.79
PDGF-bb	r_τ	-0.01	0.00	0.05	0.01	-0.03	-0.05	0.21	0.18	**0.44**	0.09	**0.44**	0.08
	p-value	0.88	0.97	0.63	0.94	0.74	0.56	0.03	0.06	**0.00**	0.37	**0.00**	0.45
RANTES	r_τ	0.01	0.00	0.04	-0.02	-0.03	-0.05	0.28	0.25	**0.43**	0.14	**0.43**	0.10
	p-value	0.90	0.97	0.68	0.89	0.79	0.60	0.00	0.01	**0.00**	0.16	**0.00**	0.33
TNF	r_τ	0.03	0.01	-0.03	0.00	0.02	-0.09	**0.40**	**0.32**	**0.57**	0.04	**0.57**	0.00
	p-value	0.72	0.92	0.78	0.97	0.80	0.37	**0.00**	**0.00**	**0.00**	0.69	**0.00**	1.00

Abbreviations: *HOA*, hand osteoarthritis; *IL*, interleukin; *INF*, interferon; *IP*, interferon gamma-induced protein; *MCP*, monocyte chemoattractant protein; *MIP*, macrophage inflammatory protein; *PDGF*, platelet-derived growth factor; *TNF*, tumour necrosis factor; *AUSCAN*, Australian/Canadian; *CRP*, C-reactive protein; *GS*, greyscale; *PD*, power Doppler.

Table S3 Osteoarthritis at additional joint sites and its influence on the levels of inflammatory mediators. The effect of OA at additional joint sites was measured in HOA patients who were divided into a) patients with hip OA; b) patients with knee OA; c) patients with both hip and knee OA; and d) patient without hip or knee OA. The LM analysis included age, gender, BMI, and CRP as confounders. *P-values* were computed using ANOVA and effect size was calculated as partial omega with 95% CI.

Mediators	p-value	F-statistics	Effect size
IFN-γ	0.211	1.541	0.019 (0 - 0.081)
MCP-1	0.310	1.212	0.006 (0 - 0.032)
IL-4	0.367	1.067	0.002 (0 - 0)
IL-17	0.416	0.962	-0.001 (0 - 0)
MIP-1α	0.427	0.935	-0.002 (0 - 0)
IL-1RA	0.542	0.721	-0.008 (0 - 0)
PDGF-bb	0.552	0.704	-0.009 (0 - 0)
TNF	0.579	0.660	-0.010 (0 - 0)
Eotaxin	0.604	0.621	-0.011 (0 - 0)
IL-1β	0.610	0.613	-0.018 (0 - 0)
IL-9	0.695	0.484	-0.016 (0 - 0)
RANTES	0.701	0.474	-0.016 (0 - 0)
MIP-1β	0.701	0.474	-0.016 (0 - 0)
IP-10	0.702	0.473	-0.016 (0 - 0)
IL-8	0.733	0.429	-0.017 (0 - 0)

Abbreviations: *HOA*, hand osteoarthritis; *IL*, interleukin; *INF*, interferon; *IP*, interferon gamma-induced protein; *MCP*, monocyte chemoattractant protein; *MIP*, macrophage inflammatory protein; *PDGF*, platelet-derived growth factor; *TNF*, tumour necrosis factor.
